# Functional analysis of alternative castor bean DGAT enzymes

**DOI:** 10.1590/1678-4685-GMB-2022-0097

**Published:** 2022-12-09

**Authors:** Thomaz Stumpf Trenz, Andreia Carina Turchetto-Zolet, Rogério Margis, Marcia Margis-Pinheiro, Felipe dos Santos Maraschin

**Affiliations:** 1 Universidade Federal do Rio Grande do Sul, Programa de Pós-graduação em Biologia Celular e Molecular, Centro de Biotecnologia, Porto Alegre, RS, Brazil.; 2 Universidade Federal do Rio Grande do Sul, Programa de Pós-graduação em Genética e Biologia Molecular, Porto Alegre, RS, Brazil.; 3 Universidade Federal do Rio Grande do Sul, Instituto de Biociências, Departamento de Genética, Porto Alegre, RS, Brazil.; 4 Universidade Federal do Rio Grande do Sul, Instituto de Biociências, Departamento de Biofísica, Porto Alegre, RS, Brazil.; 5 Universidade Federal do Rio Grande do Sul, Instituto de Biociências, Departamento de Botânica, Porto Alegre, RS, Brazil.

**Keywords:** TAG, lipids, oil, diacylglycerol acyltransferase, Ricinus

## Abstract

The diversity of diacylglycerol acyltransferases (DGATs) indicates alternative roles for these enzymes in plant metabolism besides triacylglycerol (TAG) biosynthesis. In this work, we functionally characterized castor bean (*Ricinus communis* L.) DGATs assessing their subcellular localization, expression in seeds, capacity to restore triacylglycerol (TAG) biosynthesis in mutant yeast and evaluating whether they provide tolerance over free fatty acids (FFA) in sensitive yeast. RcDGAT3 displayed a distinct subcellular localization, located in vesicles outside the endoplasmic reticulum (ER) in most leaf epidermal cells. This enzyme was unable to restore TAG biosynthesis in mutant yeast; however, it was able to outperform other DGATs providing higher tolerance over FFA. RcDAcTA subcellular localization was associated with the ER membranes, resembling RcDGAT1 and RcDGAT2, but it failed to rescue the long-chain TAG biosynthesis in mutant yeast, even with fatty acid supplementation. Besides TAG biosynthesis, our results suggest that RcDGAT3 might have alternative functions and roles in lipid metabolism.

## Introduction

Triacylglycerides (TAGs) are the main seed storage lipids in plants and are used as an energy reserve for seed germination in oleaginous plants. Besides their relevance in plant metabolism, TAGs are essential foods and raw materials for the industry (Jaworski and Cahoon, 2003; Orsavova et al,. 2015). The chemical properties of TAGs rely on their fatty acid (FA) composition and, consequently, define their industry applications (Dyer and Mullen, 2008). Plant oils are mainly composed of a mix of five FAs, such as palmitic acid (C16:0), stearic acid (C18:0), oleic acid (C18:1 Δ^9^), linoleic acid (C18:2 Δ^9,12^) and linolenic acid (C18:3 Δ^9,12,15^), which comprise the category of so-called usual fatty acids (Jaworski and Cahoon, 2003). On the other hand, some species produce distinct FAs that are rare in nature, and because of it, they are named as unusual fatty acids. These nonconventional FAs usually present functional groups (epoxy, hydroxy), shorter carbon chains, or high levels of unsaturation (Jaworski and Cahoon, 2003). 

Castor bean (*Ricinus communis* L.) seed oil contains almost 90 % of ricinoleic acid, an unusual fatty acid with a hydroxyl radical in its twelfth carbon (12-OH - C18:1 Δ^9^). Its hydroxyl group confers unique physical-chemical properties, making it more miscible in alcohol, and with high viscosity. Due to its functional group, this FA is exploited as raw material to produce plastics, paints, shampoos, cosmetics, lubricants, and other products (He et al., 2004; Shockey et al., 2019). Besides the industrial relevance of ricinoleic acid, castor bean seeds also display the ability of store TAGs with a very high content of a single FA in its oil, a rare feature to most crops (Tvrzicka et al., 2011). Nonetheless, the biochemical pathways that lead castor bean to be able to produce TAGs with a very high content of a single FA remain unclear, although a co-evolution of its enzymes should be considered (Burgal et al., 2008; Shockey *et al.*, 2019).

In plants, two enzymes are responsible for catalyzing the formation of TAGs: Phospholipid:diacylglycerol acyltransferase (PDAT), which uses phospholipids and diacylglycerol (DAG) as substrates, and diacylglycerol acyltransferase (DGAT), which catalyzes the acylation of acyl-CoA into the *sn*-3 position of DAGs, resulting in the formation of TAGs. The latter is considered the main enzyme for oil formation (Maraschin et al., 2019; Turchetto-Zolet et al., 2011). There are at least five types of DGATs, named as DGAT1, DGAT2, DGAT3, DAcT, and WS/DGAT. DGAT1 is the most well-characterized in animals and plants. In mammals, this enzyme is localized in the membranes of the endoplasmic reticulum (ER), and it is suggested to have many acyltransferase activities other than the acylation of DAG, such as acyl-CoA:retinol acyltransferase and monoacylglycerol acyltransferase (Yen et al., 2008). In plants, DGAT1 has an expression profile wider than the other DGAT isozymes, being expressed in several tissues, such as flowers, leaves, shoots, and seeds (Cao et al., 2013; Chen et al., 2007; Chen *et al.*, 2016). DGAT2 is also well-described, with homologs found also in fungi (Sandager et al., 2002). Plant DGAT2 is highly expressed during seed development in many species, especially in plants bearing unusual fatty acids (FA) (Burgal et al., 2008; Cao *et al.*, 2013; Kroon et al., 2006). DGAT2 is localized in different ER subdomains compared to DGAT1, indicating their functions are nonredundant (Shockey et al., 2006).

Several attempts to increase unusual FA content in *Arabidopsis thaliana* through heterologous expression have been tested, and although significant results were achieved, they were far from mimicking the high content found in the original species (Burgal et al., 2008; Lee et al., 1998; van Erp et al., 2011; Yurchenko et al., 2017). Heterologous expression of the fatty acid Δ12-hydroxylase (RcFAH12) in the *fatty acid elongase1* (*fae1*) mutant background yielded Arabidopsis seeds with ~17% of hydroxy-fatty acids (HFAs). Later, these *fae1*:RcFAH12 lines (named as CL37) were used to either express RcDGAT2 or RcPDAT1, increasing the ricinoleic acid content to almost 20 %, and HFAs in ~30% in Arabidopsis seeds (Burgal *et al.*, 2008; Kim et al., 2011; van Erp *et al.*, 2011). A recent attempt of co-expressing three castor bean acyl-transferases in the CL37 lines achieved even higher levels (~35%) of HFAs in *A. thaliana* oil (Lunn et al., 2019). However, it was still distant from the 90 % found in castor bean seeds. Therefore, other enzymes might be related to the accumulation of the unusual FA in lipid droplets and their removal from cell membranes (Lunn *et al.*, 2020).

New enzymes related to the biosynthesis of oil displaying DGAT activity were described in the past few years. The wax ester synthase/acyl coenzyme A:diacylglycerol acyltransferase (WS/DGAT) is a bifunctional enzyme that exhibits both acyl-CoA:fatty acid acyltransferase and DGAT activities. It is present in prokaryotes (Arabolaza et al., 2008; Kalscheuer and Steinbüchel, 2003) and it was also found and characterized in *Arabidopsis thaliana* (Li et al., 2008). Another enzyme different from DGAT1 and DGAT2 was identified, which is responsible for producing acetyl-triacylglycerides (acTAGs), abundant in *Euonymus alatus* seeds (Durrett et al., 2010). This distinct TAG has interesting properties due to its low viscosity, and it could be used in the biodiesel composition, avoiding the transesterification process. The enzyme identified was named as diacylglycerol acetyltransferase (DAcT), which adds acetyl at the *sn*-3 position of a DAG. Beyond this activity, EaDAcT can also acetylate fatty alcohols *in vitro* (Bansal and Durrett, 2016). Heterologous expression of this enzyme in Arabidopsis yielded 40% of acTAGs in its oil, showing an attractive application of DAcT in the formation process of low viscosity oils for the production of biofuels (Durrett *et al.*, 2010; Liu et al., 2015; Tran et al., 2017a); however, no homologous DAcT was identified and characterized for oilseed crops, or plants with relevant oil in industry applications (Alkotami et al., 2021; Mihálik et al., 2020; Tran *et al.*, 2017b).

A soluble DGAT, named DGAT3, was identified in the cytosolic fractions of developing cotyledons of peanut (*Arachis hypogaea*) (Saha et al., 2006). DGAT3 possesses a low identity compared to other DGATs, and it does not contain any transmembrane domains. Later, a truncated version of *Arabidopsis thaliana* DGAT3 was expressed in protoplasts and displayed a cytoplasmic subcellular localization, remarkably different from the ER subcellular localization found for AtDGAT1 (Hernández et al., 2012). Furthermore, AtDGAT3 contains a thioredoxin-like ferredoxin domain that has been shown to bind to [2 Fe-2 S] cluster (Aymé et al., 2018). Also, DGAT3 homologs are highly expressed in leaves in contrast to other DGATs (Cao et al., 2013; Turchetto-Zolet et al., 2016). Even with the recent progress in the functional characterization of AtDGAT3 and AhDGAT3-3 enzymes, there are still many questions regarding the soluble DGATs and their role in lipid metabolism of plants (Aymé *et al.*, 2018; Chi et al., 2014).

This work focused on the characterization of the alternative DGATs enzymes DGAT3 and DAcT from castor bean (*Ricinus communis*), centering on the highly expressed DGAT3, aiming to understand their role in lipid metabolism. Our work provides evidence that the RcDGAT3 shows distinct subcellular localization and enzymatic properties from other DGATs, pointing to new functions for DGAT3 in plant metabolism.

## Material and Methods

### Identification of castor bean *DGAT3* and *DAcT*

To identify the castor bean *DAcT* and *DGAT3* sequences, a systematic search was performed at Castor Bean Genome Annotation (http://castorbean.tigr.org/), using *EaDAcT* gene from *Euonymus alatus* (GenBank: GU594061), and *AhDGAT3* gene from peanut (*Arachis hypogaea*, GenBank: AAX62735) as queries. BLAST (Basic Local Alignment Search Tool) was used to search for the putative DGAT genes, in its tBLASTx configuration. Selected sequences, which had an E-value lower than 10^-50^, were compared to previously characterized sequences of *DAcT*, or *DGAT3*, using *Arabidopsis thaliana* (https://www.arabidopsis.org//) and Phytozome (http://www.phytozome.net/) databases, to identify the coding genes of DAcT and DGAT3 in castor bean. Transmembrane domains were predicted by DeepTMHMM (Hallgren et al., 2022). Protein domains were predicted on Conserved Domains Database (CDD, NCBI).

### Gene expression during castor bean seed development

**Figure 1-:**
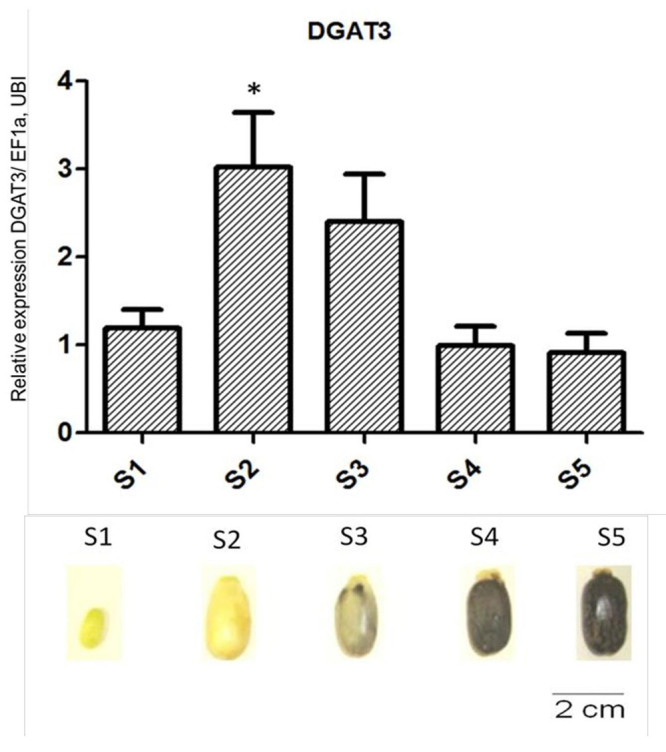
*RcDGAT3* mRNA expression profile in castor been (*Ricinus communis* L.) developing seeds. Relative RT-qPCR expression using stage S1 as reference and *RcUBI* and *RcEF1a* as reference genes. Developing seed stages (S1 - S5) were described in (Cagliari et al., 2010). Bars represent standard error, and the asterisk indicates P < 0.05 by ANOVA test.

Castor bean seed cDNA from commercial AL-Guarani variety was previously available (Cagliari et al., 2010) and it was used to evaluate the gene expression of five different development stages of castor bean seeds based on morphological characteristics (such as color, texture and, size, Figure 1). Evaluation of gene expression was performed by RT-qPCR as described. Gene-specific synthetic oligonucleotides were designed (Table S1) using Primer3 software (http://frodo.wi.mit.edu/primer3/primer3_code.html). Expression of ubiquitin (RcUBI) and Elongation factor 1-α (RcEF1α) were used as references (Cagliari *et al.* 2010). Five biological replicates were used, and three technical replicates were performed for each reaction. SYBR-Green fluorescence was analyzed by StepOne software version 2.1 (Applied Biosystems), and the Cycle Threshold (CT) value for each sample was calculated and reported using the 2^-ΔΔCT^ method (Livak and Schmittgen, 2001). Statistical significance was tested by Analysis of Variance (ANOVA), followed by Dunnett, comparing every stage to the first stage, S1.

### Plasmid construction

Full-length *RcDGAT3* (XM_002519293) and *RcDAcTA* (XM_002528977) CDS were amplified from castor bean leaf cDNA, using gene-specific primers (Table S2), different reverse primers contained or not the stop codon, to allow gene fusions to fluorescent tags. Amplicons were cloned into pENTR/D-TOPO vector to generate Gateway entry clones (Invitrogen). For expression in *Saccharomyces cerevisae*, *RcDGAT3* and *RcDAcT* amplified by primers Rc_DGAT3_TOPOf and Rc_DGAT3_STOPr were subcloned from pENTR to pVT-U103 (Vernet et al., 1987), using BamHI and XbaI restriction sites. Expression plasmids pVT-U103 carrying *RcDGAT1* CDS, or *RcDGAT2* CDS were previously described (Turchetto-Zolet et al., 2011). pVT-U103 vector contains the alcohol dehydrogenase I (*ADH1*) constitutive promoter that drives the heterologous expression of DGAT genes. Entry vectors in pENTR/D-TOPO carrying *RcDGAT1*, *RcDGAT2*, *RcDGAT3*, or *RcDAcTA* were used in a LR Clonase™ (Invitrogen) reaction with pART7gateway-YFP:HA to generate translationally fused proteins with the Yellow Fluorescent Protein (YFP). The same entry vectors carrying *RcDGAT1* and *RcDGAT2* CDS were recombined with pEARLYGATE-103 (Earley et al., 2006) to generate translationally fused proteins with the Green Fluorescent Protein (GFP). pENTR_RcDGAT3 was recombined with the binary vector pH7CWG2 (Karimi et al., 2005), to generate a construction carrying RcDGAT3 translationally fused with the Cyan Fluorescent Protein (CFP).

### Yeast growth, mutant complementation, and lipotoxicity assays

H1246 mutant yeast strain (*Saccharomyces cerevisiae*), unable to synthesize TAG due to mutations on *ARE1/ARE2/LRO1/DGA1* genes (Sandager et al., 2002), was transformed as previously described (Turchetto-Zolet et al., 2011) with RcDGAT1, RcDGAT2, RcDGAT3, or DAcTA expression cassettes for phenotype complementation test. In parallel, wild type G175 and mutant H1246 strains were transformed with empty vectors and used as positive and negative controls, respectively. Yeast cultures were grown at 30 °C for 72 hin minimum media containing 0.67% of Yeast Nitrogen Base without amino acids (Merck), 2% of glucose and amino acids drop out lacking uracil. Cells were harvested and washed three times with 0,9% (w/v) NaCl and resuspended in 1 ml of the same solution. Cells were homogenized with the same volume of glass beads (0.5 mm), with intense vortex for five min. Lipids were extracted with chloroform /methanol /0.9% NaCl in water solution (2:1:1, v/v/v). The organic phase was collected, dried, and resuspended in chloroform. Lipids were applied on silica gel plate for thin layer chromatography (Pan et al., 2013), using hexane/diethyl ether /acetic acid (80:20:1, v/v/v) as mobile phase. Lipids were visualized using iodine vapor. Soybean oil was used as a TAG reference.

For complementation assays in yeast using supplementation with linoleic and linolenic acids (Sigma), the fatty acids were first dissolved in ethanol to a concentration of 0.5 M. The FA solutions were then dissolved in 0.05% Triton X-100 in ethanol and directly added to the medium. Yeast suspensions were diluted to an initial OD of 0.1, supplemented with 0.2 mM of linoleic acid or linolenic acid, grown for 72 h at 30 °C, and their lipids were extracted and evaluated as described above.

For the lipotoxicity assay, yeast cultures were grown in medium without fatty acid supplementation until an OD of 2.2 ± 0.2. Later, 10 µl from the different cultures were added to the plates containing linoleic acid, or linolenic acid, with different concentrations (0.1 mM, 0.5 mM, and 1.0 mM). Serial dilutions of 10^-1^, 10^-2^, 10^-3^, 10^-4^ were prepared from yeast cultures, and 10 µl of each dilution was applied on the plates containing the fatty acids. H1246 yeast strain carrying the empty vector (pVT-U103) was used as the negative control. The plates were kept at 30 °C for seven days before imaging.

### Nile red assay

Yeast cultures were grown to the stationary phase (72 h), and Nile red assay was performed as previously described (Siloto et al., 2009).

### Transient expression of fluorescent-tagged fusion proteins

Protoplasts from *Arabidopsis thaliana* mesophyll cells were obtained through the Tape-*Arabidopsis* Sandwich method (Wu et al., 2009) and transformed as described previously (Yoo et al., 2007). Protoplasts were transformed with expression plasmids carrying castor bean *DGAT* genes (pART7_DGAT1-YFP:HA, pART7_DGAT2-YFP:HA, pART7_DGAT3-YFP:HA, or pART7_DAcTA-YFP:HA) co-transformed with pB7WGR2-RNTLB13 binary vector. The Reticulon-Like Protein B13 (RNTLB13) is an endoplasmic reticulum protein and is translationally fused with a red fluorescent protein (RFP) (Sparkes et al., 2010). For transient expression in *Nicotiana benthamiana* leaves, plants were grown at 24 °C, with a photoperiod of 16 h: 8 h of light: dark for 45 days, until leaves were fully expanded for agroinfiltration, which was performed as described previously (Sparkes *et al.*, 2006). *Agrobacterium tumefaciens* (LBA4404 strain) cell suspensions carrying either pEARLYGATE-103_RcDGAT1, pEARLYGATE-103_RcDGAT2, or pH7CWG2_RcDGAT3 binary vectors were co-infiltrated with suspensions carrying the pB7WGR2-RNTLB13 binary vector (Sparkes *et al.*, 2010), in an optical density ratio of 2:1. Transient expression of fluorescent proteins was visualized via confocal fluorescence microscopy in an Olympus FV1000 confocal laser scanning microscope.

### *Arabidopsis thaliana* transformation

*A. thaliana* Col-0 plants were grown for 40 days, at 24 °C, with a photoperiod of 16 h : 8 h of light : dark, and transformed with *Agrobacterium tumefaciens* (LBA4404 strain) carrying pH7CWG2_DGAT3 via the floral-dip method (Zhang et al., 2006). T1 plants were selected in medium containing hygromycin (25 mg/l), carbenicillin (500 mg/l) and nystatin (50 mg/l), and DGAT3-CFP transgene was confirmed by PCR with Rc_DGAT3f and Rc_DGAT3r primers. T3 generation homozygous plants were visualized by confocal fluorescence microscopy.

## Results

### DGAT3 is expressed in castor bean developing seeds

The coding sequences of DGAT3 and DAcT were searched in the castor bean genome revealing the presence of one homologous sequence to *AhDGAT3*, and four putative coding genes for DAcT named as *DAcTA, DAcTB, DAcTC, DAcTD* (Table 1).

**Table 1-:** *DGAT1* , *DGAT2* and homologous genes of EaDAcT and AhDGAT3 identified in castor bean (*Ricinus communis* L.).

Gene	Access^*^	AA	Exons	Introns	TMH	Predicted Domains
*DGAT1*	29912.m005373	521	9	8	9	DGAT, MBOAT
*DGAT2*	29682.m000581	340	5	4	2	DGAT, LPLAT
*DGAT3*	29889.m003411	332	2	1	0	TRX_Fd
*DAcTA*	27613.m000613	359	1	0	8	MBOAT
*DAcTB*	27613.m000612	406	6	5	8	MBOAT
*DAcTC*	29812.m000198	369	1	0	8	MBOAT
*DAcTD*	29990.m000512	367	1	0	8	MBOAT

^*^Access codes can be used in the JGI - Phytozome database.

AA = Aminoacids; TMH = Transmembrane helices; DGAT = Diacylglycerol acyltransferase; MBOAT = Membrane bound O-acyl transferase family; LPLAT = Lysophospholipid acyltransferase; TRX_Fd = Thioredoxin (TRX)-like [2Fe-2S] Ferredoxin (Fd) family.

To verify whether the *DGAT3* and the four *DAcT* putative genes are expressed in seeds, the steady-state mRNA of five seed developing stages, named as S1, S2, S3, S4 and, S5 as previously described (Cagliari et al., 2010), was quantified via RT-qPCR. Expression of the four putative *DAcT* genes was not detected in castor bean seeds (data not shown); however, *DGAT3* expression was observed throughout seed development, with maximum expression at stage S2, which represents the total seed expansion and high carbon mobilization for TAG synthesis (Cagliari *et al.*, 2010) (Figure 1). These results agree with *DGAT3* expression levels found in the castor bean transcriptome (Brown et al., 2012), which reveals that *DGAT3* is more expressed in the early stages of endosperm development as well as highly expressed in leaves and flowers (Figure S1). Although we did not detect expression of any *DAcTs* genes during the seed development, *RcDAcTA* was selected as the only castor bean DAcT to be further characterized, since it displays the most similar sequence to DAcT from *Euonymus alatus*, and it also showed a low but detectable level of expression in the same transcriptomic database (Brown *et al.*, 2012) (Figure S1).

### DGAT3 has a distinct subcellular localization compared to other DGATs

**Figure 2 -:**
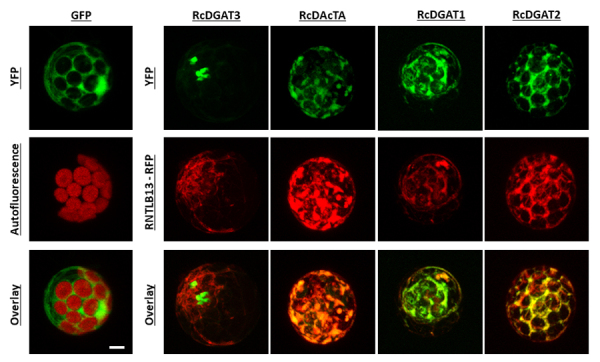
Subcellular localization of castor bean acyltransferases. Protoplasts from Arabidopsis mesophyll cells transiently expressing GFP, RcDGAT3-YFP, RcDAcTA-YFP, RcDGAT1-YFP, or RcDGAT2-YFP. The left panel shows the fluorescence of GFP alone and the chloroplasts autofluorescence (red), as well as the overlay of both images. The right panel shows the fluorescence of RcDGAT proteins translationally fused with YFP, the fluorescence of the endoplasmic reticulum protein marker RNTLB13-RFP (in red), and the overlay of both images (last line). Scale bar = 5 µm.

**Figure 3 -:**
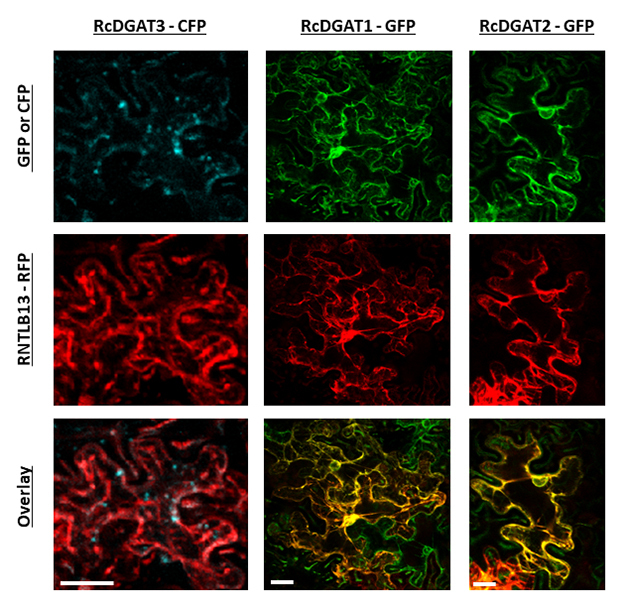
Transient expression of castor bean DGATs in *Nicotiana benthamiana* leaves. *N. benthamiana* leaves were co-infiltrated with *Agrobacterium tumefaciens* carrying the DGATs CDS translationally fused with CFP, or GFP and with *Agrobacterium tumefaciens* carrying the RTNLB13 (ER marker) CDS translationally fused with RFP. The first row displays the CFP (blue), or GFP (green) fluorescence; the second row displays the fluorescence of RTNLB13-RFP (red); and the last row shows the overlay of both images. Scale bars = 20 µm.

Most DGAT enzymes are located in the endoplasmic reticulum (Shockey et al., 2006; Chen et al., 2016). To assess where castor bean DGAT proteins are located inside cells, Arabidopsis mesophyll protoplasts were transfected with DNA constructs of *RcDGATs* and *RcDAcTA*, translationally fused with YFP, and co-transfected with the ER marker RTNLB13-RFP (Shockey *et al.*, 2006). DGAT1 and DGAT2 were localized in the ER, as well as DAcTA (Figure 2); DGAT3, however, showed a different subcellular localization, not associated with the endoplasmic reticulum (Figure 2). To confirm the subcellular localization of DGAT3 *in planta,* we evaluated *N. benthamiana* leaves co-agroinfiltrated with RNTLB13-RFP. DGAT1 and DGAT2 were observed associated with the ER membranes co-localized with the ER-marker, whereas DGAT3 was mainly visible as dot-like structures inside the cells (Figure 3). These results show that castor bean DGAT1, DGAT2, and DAcTA are endoplasmic reticulum proteins, and DGAT3 displays a different subcellular localization.

### Castor bean DGAT3 abundance might be post-translationally regulated

**Figure 4-:**
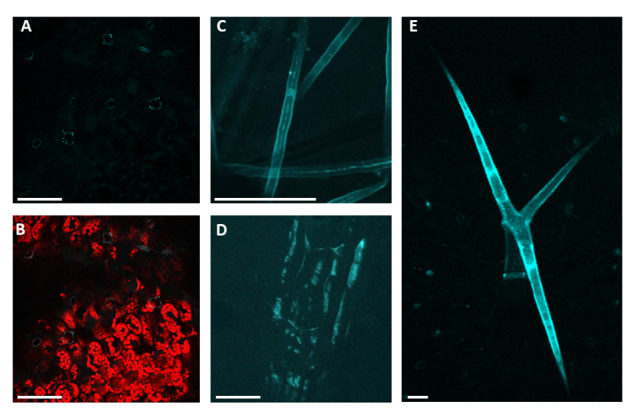
RcDGAT3 accumulates in A. thaliana epidermal cells. Confocal fluorescence microscopy images from different tissues of transgenic A. thaliana plants expressing 35S::DGAT3-CFP. (A) Arabidopsis leaf; (B) Same image as (A) with overlaid chlorophyll autofluorescence; (C) Root hairs and (D) Root epidermis, (E) Trichome. Scale bars = 50 µm.

Protein function is associated with its subcellular localization, and it can vary due to the distinct cellular environments found for each type of cell. To assess the castor bean DGAT3 cellular localization in stably transformant plants, *A. thaliana* plants were transformed with *DGAT3* CDS translationally fused with CFP, and its expression was driven by the 35S promoter (Figure 4 and Figure S2). Interestingly, the stably transformed plants presented a restricted fluorescence pattern for the DGAT3-CFP fusion protein. In stomatal guard cells, DGAT3-CFP was localized in dot-like structures resembling vesicles (Figure 4A) consistent with the transiently expressed protein in protoplasts and agroinfiltrated *N. benthamiana* leaves (Figures 2 and 3). Moreover, CFP fluorescence was absent from leaf mesophyll cells (Figure 4B) as well as most of the vegetative tissues in the transgenic lines. Conversely, DGAT3-CFP fluorescence was identified throughout the cytoplasm in epidermal tissues such as root hairs, root epidermis and trichomes (Figure 4C-E, respectively), suggesting its protein accumulation might be suppressed post-translationally except for epidermal tissues.

### DGAT3 and DAcTA are unable to rescue the TAG biosynthesis in mutant yeast

**Figure 5-:**
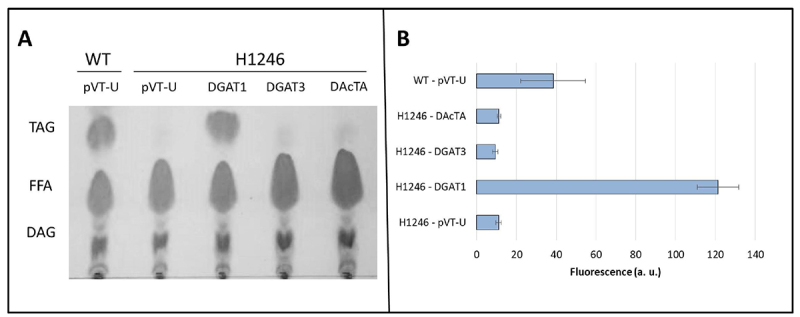
Complementation assay using mutant yeast unable to produce oil. (A) Thin-layer chromatography (TLC) of total lipid extract of wild type yeast (WT) or TAG synthesis mutant yeast (H1246). Cells were transformed with empty pVT-103U (empty vector), or with the plasmid containing castor bean *DGAT1*, *DGAT3* or *DAcTA* CDS. Cells were grown in a minimum medium without uracil for 72 hours. (B) *In vivo* DGAT activity using Nile red stain in mutant yeast. TAG: Triacylglycerides; FFA: Free Fatty Acids; DAG: Diacylglycerol.

Diacylglycerol acyltransferases can be considered the limiting enzymes for TAG production. A yeast mutant complementation assay was performed to assess the function of the putative *DGAT3* and *DAcTA* genes, using H1246 mutant strain which lacks all DGAT related activity in yeast (Sandager et al., 2002) (Figure 5). The yeast cells were transformed with expression vectors for the constitutive expression of the coding sequences of RcDGATs. The transformed cells were grown until their steady-state phase, which is the phase where yeast can accumulate TAGs. The heterologous expression of both castor bean DGAT3 and DAcTA was unable to rescue triacylglycerol synthesis (Figure 5A). To further verify whether the castor bean genes could produce neutral lipids, the Nile red fluorimetric assay was performed *in vivo* (Figure 5B). Neither the fluorescence of mutant cells expressing DGAT3 nor those expressing DAcTA had significant differences to the fluorescence of H1246 cells containing the empty vector (Figure 5B). We also did not observe complementation with the putative *DAcT* soybean genes *Glyma13g17860* and *Glyma17g04650* (data not shown). These results indicate that castor bean *DGAT3* and *DAcTA* genes cannot rescue TAG biosynthesis in mutant yeast.

### DGAT3 can overcome lipotoxicity caused by FFA in yeast without producing TAGs

**Figure 6-:**
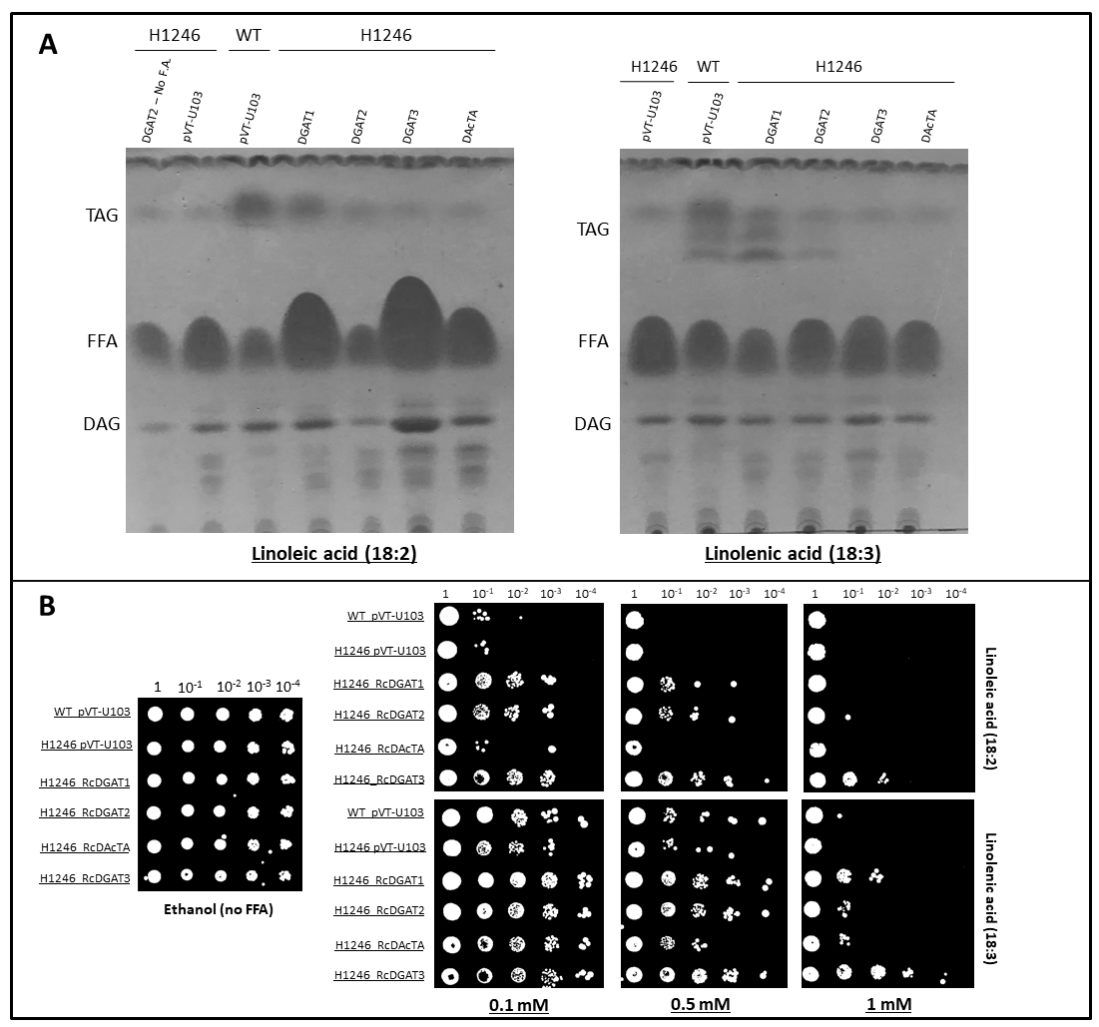
Complementation assay using H1246 mutant yeast and free fatty acid (FFA) supplementation. (A) TLC from lipid extracts of WT yeast and mutant yeast (H1246) carrying the empty vector (pVT-U103), or expressing different castor bean DGATs (RcDGAT1, RcDGAT2, RcDGAT3 and RcDAcTA). Yeast were grown in the presence of 0.2 mM of linoleic acid (18:2, left panel), or linolenic acid (18:3, right panel). (B) Rescue of lipotoxicity phenotype in H1246 cells expressing RcDGAT genes. Yeast were grown for seven days in selective medium (without uracil), in the absence of FFA (left panel), in the presence of linoleic acid (18:2, right panel, first line), or linoleic acid (18:3, right panel, second line) in different concentrations (0.1, 0.5 and 1 mM). Yeast growth is displayed by applying 10 μl of each dilution (1 to 10^-4^, from left to right) of mutant (H1246) or WT yeast. TAG: Triacylglycerides; FFA: Free Fatty Acids; DAG: Diacylglycerol.

DGAT3 displays a distinct subcellular localization (Figures 2, 3 and 4) and it is also more expressed in leaves and male flowers (Figure S1), suggesting it has a different role in lipid metabolism than the other DGATs. For this purpose, we selected two fatty acids commonly found in castor bean pollen, flowers and also in plant leaves (Li-Beisson et al., 2010; Brown et al., 2012), to supplement the culture media of TAG-deficient H1246 mutant yeast cells carrying vectors to express *DGAT* genes. Both linoleic (C18:2) and linolenic (C18:3) acids are not produced by H1246 mutant yeast cells (Sandager et al., 2002), and we hypothesized that the castor bean enzymes would require specific substrates for mutant yeast complementation. Regarding the supplementation with linoleic acid, only the wild type (WT) yeast and the H1246 yeast expressing DGAT1 were able to produce triacylglycerides (Figure 6A); however, when these cells were supplemented with linolenic acid, DGAT2-expressing mutant cells were also able to produce TAGs, besides the WT and DGAT1-expressing mutant cells (Figure 6A). Both H1246 strains carrying DGAT3, or DAcTA CDS did not produce detectable triacylglycerides, even with the supplementation of polyunsaturated fatty acids (Figure 6A).

Free fatty acids are toxic for H1246 mutant yeast due to their inability to convert them to less reactive compounds like TAGs (Pan et al., 2013). The ability to rescue yeast growth in medium containing exogenously supplied fatty acids can be used to evaluate DGAT activity towards the conversion of FA to TAG. Initially, wild-type and mutant yeast cells were plated in serial dilutions in a selective medium to assess the standard cell growth in FFA free medium (Figure 6B, left panel). To evaluate the effects caused by linoleic acid and linolenic acid, these fatty acids were added to the media in three concentrations (0.1, 0.5, and 1 mM). Overall, linoleic acid displayed a more significant inhibitory effect than linolenic acid, regardless of the genotype or concentration used (Figure 6B). WT yeast, carrying the empty vector, was able to tolerate low concentrations of both fatty acids, displaying slightly reduced growth. On the other hand, in higher concentrations, the WT yeast was not able to grow in higher cell dilutions, indicating a lipotoxic effect caused by the supplementation of FFA (Figure 6B, right panel). Mutant yeast (H1246) expressing castor bean RcDGAT1 grew better than the mutant and the WT in the presence of linolenic acid, when compared to their respective empty vector controls in free of FFA medium (Figure 6B, left and right panels), indicating that RcDGAT1 can detoxify linolenic acid. For linoleic acid, RcDGAT1-expressing mutant yeasts were able to grow only in low concentrations, indicating that RcDGAT1 is able to detoxify linoleic acid to some extent. RcDGAT2-expressing mutant yeast was only able to grow in low concentrations of linoleic and linolenic acids, suggesting that DGAT2 enzyme can detoxify these FFA to a lower extent compared to RcDGAT1 (Figure 6B). Surprisingly, RcDGAT3-expressing mutant yeast cells grew better in the presence of either linoleic acid or linolenic acid, compared to the other mutant and wild-type yeasts. Only the highest concentration of linoleic acid was able to substantially reduce the growth of H1246 cells expressing DGAT3 (Figure 6B, right panel). This phenotype supports that RcDGAT3 is expressed in the yeast cells and, although it does not complement TAG biosynthesis, it confers a detectable phenotypic alteration. On the other hand, RcDAcTA-expressing mutant yeast growth was higher than the empty vector control only in lower concentrations of linolenic acid. These results suggest that, although unable to produce detectable levels of TAG in mutant yeast, RcDGAT3 shows higher activity towards detoxifying unsaturated fatty acids than DGAT1 and DGAT2.

## Discussion

Diacylglycerol acyltransferases are the main enzymes in TAG biosynthesis in most organisms, and they have been explored for biotechnological use to improve oil production (Reynolds et al., 2017). Although the heterologous expression of DGAT1 and DGAT2 has shown the ability to redirect the lipid metabolism to the anabolism of TAGs, many mechanisms remain unclear. The co-expression of DGAT and specific fatty acid desaturases/hydrolases to produce TAGs with unusual fatty acids, were shown to yield low levels of these molecules, which limits their use in substitution for oil extraction from non-crop oilseeds as castor bean (Burgal et al., 2008; Yurchenko et al., 2017). 

Castor bean *DGAT1* and *DGAT2* are expressed in seeds, with the latter being more actively expressed throughout seed development (Cagliari et al., 2010). *DGAT3* is also expressed during seed development (Figure 1). Likewise, soybean *DGAT3* genes were found to be expressed during seed development (Turchetto-Zolet et al., 2016), indicating that this gene might be related to seed lipid metabolism. Furthermore, based on a castor bean transcriptome (Brown et al., 2012), *RcDGAT3* is highly expressed in leaves and male flowers (Figure S1). This pattern was also observed in tung trees (*Vernicia fordii*), in which *DGAT3* is more expressed in flowers and leaves than seeds (Cao et al., 2013). In addition, castor bean DGAT3 displays a distinct subcellular localization than the other DGAT proteins, which might be associated with its function (Figure 4). Hernández and colleagues proposed that soluble DGAT may be related to the management of the acyl-pool and its composition in response to the membrane lipid biosynthesis demand (Hernández *et al.*, 2012). 

Despite the nonredundant functions, we demonstrated that both castor bean DGAT1 and DGAT2 are attached to the endoplasmic reticulum membranes (Figures 2 and 3), similarly what was shown for homologous of these enzymes in other species (Shockey et al., 2006; Chen et al., 2016); however, although the absence of transmembrane domains, the DGAT3 subcellular localization is still controversial. *A. thaliana* DGAT3 was first shown to be cytosolic (Hernández et al., 2012). Later, its sequence annotation was revised. It became clear that the translated sequence used for the subcellular localization prediction lacked the first 75 codons, which indicated a putative transit peptide to the chloroplast (Aymé et al., 2018). Castor bean DGAT3 also contains this N-terminal peptide (Aymé *et al.*, 2018), but in our work conditions, its presence was not observed in the chloroplast but rather in cytoplasmic “dot-like” structures. Our results suggest that this enzyme accumulates in epidermal tissues (Figure 4) and stomatal guard cells (Figure 2, 3 and 4). Interestingly, Arabidopsis plants overexpressing RcDGAT3-CFP by the constitutive 35S promoter only accumulated the recombinant protein in epidermal cells, suggesting some post-translational regulation of RcDGAT3 might occur in non-epidermal tissues. Lipid droplets are present in guard cells, and their localization is similar to the pattern observed for DGAT3-CFP. Besides, their catabolism is one of the main driving forces that lead to stomatal opening (McLachlan et al., 2016). Considering the subcellular localization pattern we found for RcDGAT3, it is feasible to speculate its association with lipid droplets in guard cells, although further experiments are needed to support that.

*Euonymus alatus* DAcT was previously expressed in yeast, and *in vitro* experiments indicated that this enzyme has an endoplasmic reticulum subcellular localization (Tran et al., 2017b). Here, we studied one putative castor bean RcDAcTA and showed its ER subcellular localization in plant cells (Figure 2); however, the expression of the four putative castor bean *RcDAcT* genes was not detected throughout seed development, suggesting they do not play a major role in this organ. Besides, RcDAcTA failed to rescue the long-chain TAG synthesis in mutant yeast (Figure 5). Except for a small detoxification activity when supplied with linolenic acid (Figure 6A). These results indicate that castor bean *DAcT* genes might have other biochemical functions, as such, the already described acetylation of unsaturated DAG and fatty alcohols (Bansal and Durrett, 2016). Besides, they are phylogenetically distant from the *Euonymus* homologues (Figure S3). Therefore, further studies should be performed to unveil the role of these enzymes in long-chain TAG accumulative species.

The biochemical functions of DGAT1 and DGAT2 have been described and reviewed for many organisms (Yen et al., 2008; Maraschin et al., 2019). DGAT2 activity seems to be the major contributor for the accumulation of unusual FA in oilseeds, whereas DGAT1 seems to be more broadly expressed in other tissues and able to use common fatty acids (Kroon et al., 2006; Burgal et al., 2008; Cao et al., 2013). The heterologous expression of *Ricinus communis* DGAT2 in H1246 mutant yeast was unable to recover the TAG biosynthesis (Turchetto-Zolet et al., 2011), and the recombinant protein was only used to perform *in vitro* experiments using exogenous DAG, such as diricinolein, as substrate (Burgal *et al.*, 2008). Here, H1246 cells expressing RcDGAT2 were able to recover TAG synthesis when linolenic acid was added to the medium (Figure 6 A), demonstrating that RcDGAT2 is also able to use yeast endogenous DAG as a substrate when supplied with linoleic acid. This result agrees with those from Regmi et al. (2020), which suggest that RcDGAT2 might have a higher selectivity towards linolenic acid than other DGAT2 homologs (Regmi *et al.*, 2020).

The expression of RcDGAT1 by mutant yeast shows its ability to rescue the TAG synthesis even without FFA supplementation, as observed for its *Brassica napus* homologs (Siloto et al., 2009). Yeast cells expressing RcDGAT1 were able to tolerate high levels of linoleic and linolenic acids, converting them into TAGs (Figure 6A and B). Similar results were observed with *Linum usitatissimum* DGAT1 when linolenic acid was added to the medium (Pan et al., 2013). Our results indicate that castor bean RcDGAT1 significantly improves the tolerance to the lipotoxic effect caused by FFA through the condensation of these molecules in TAGs. RcDGAT2 was also able to do it, but to a lesser extent than RcDGAT1 (Figure 6A and B). On the other hand, mutant yeast expressing RcDGAT3 were unable to rescue TAG biosynthesis (Figure 5). The supplementation of linoleic acid, or linolenic acid to the medium was insufficient to convert them into TAGs (Figure 6A). Gao et al. (2021) showed that the expression of *Camelina sativa* DGAT3-3 in H1246 yeast cells greatly increases the TAG content only when additional substrates are added to the media. It might indicate that a different set of FAs or DAGs are needed for RcDGAT3 produces detectable TAG levels. Conversely, the expression of RcDGAT3 allowed H1246 cells to tolerate high levels of linoleic and linolenic acid regardless of TAG synthesis, which indicates that RcDGAT3 might have a distinct function other than diacylglycerol acyltransferase (Figure 6B). Soluble DGATs from plants contain a thioredoxin-like ferredoxin domain that is able to bind to [2 Fe-2 S] cluster and it has been suggested to be associated with a putative desaturase activity of DGAT3 (Aymé et al., 2018), due to the increase of C18:2 and C18:3 species in TAGs in *N. benthamiana* leaves expressing a truncated version of AtDGAT3 (Hernández et al., 2012). However, the recombinant truncated versions of AtDGAT3, lacking the N-terminal transit peptide domain, were also unable to produce TAGs *in vitro* (Aymé *et al.*, 2018). Biochemical experiments using recombinant peanut AhDGAT3-1 have shown an acyl-CoA hydrolase activity with later DGAT activity (Saha et al., 2006). Also, another homolog of AhDGAT3-1 (AhDGAT3-3) was able to restore the TAG biosynthesis in yeast mutant (Chi et al., 2014). Notwithstanding, both peanut soluble DGATs contain important residues in their DGAT1-like and GPAT-like motives absent in castor bean DGAT3 (Aymé *et al.*, 2018), which may lead to different functions of these homologs. To this purpose, new sets of substrates should be used to reveal the biochemical activity of RcDGAT3, as well as protein-protein interactions and post-translation modifications studies to unveil the importance of its subcellular localization and its role in lipid metabolism.

In conclusion, our work describes a putative DGAT (RcDGAT3) that displays distinct features from other diacylglycerol acyltransferases. RcDGAT3 does not have any transmembrane domains (Turchetto-Zolet et al., 2016), and is localized in the cytoplasm in most tissues. However, it has a vesicular localization in guard cells, leaf epidermal tissue, and mesophyll protoplasts. Also, whereas not producing TAGs in mutant yeast, RcDGAT3 was able to outperform RcDGAT1 and RcDGAT2 on recovering the lipotoxic effect caused by the addition of free fatty acids in the medium. Our results indicate that RcDGAT3 is not a *bonafide* diacylglycerol acyltransferase enzyme, but displays higher detoxifying properties than its homologs which point to new functions for DGAT3 in castor bean.

## Supplementary material

The following online material is available for this article:

Table S1 - Primers used for RT-qPCR

Table S2 - Primers used for cloning castor bean (*Ricinus communis* L.) *DGAT3* and *DAcTA* CDS

Figure S1 - Transcripts Per Kilobase Million (TPM) of castor bean *DGAT* genes in different tissues

Figure S2 - Relative expression of RcDGAT3-CFP in transgenic T3 *A. thaliana* seedlings

Figure S3 - Maximum Likelihood tree using castor bean putative *DAcT* CDS sequences and other acetyl-TAG producing plant sequences

## References

[B1] Alkotami L, Kornacki C, Campbell S, McIntosh G, Wilson C, Tran TNT, Durrett TP (2021). Expression of a high-activity diacylglycerol acetyltransferase results in enhanced synthesis of acetyl-tag in camelina seed oil. Plant J.

[B2] Arabolaza A, Rodriguez E, Altabe S, Alvarez H, Gramajo H (2008). Multiple pathways for triacylglycerol biosynthesis in Streptomyces coelicolor. Appl Environ Microbiol.

[B3] Aymé L, Arragain S, Canonge M, Baud S, Touati N, Bimai O, Jagic F, Louis-Mondésir C, Briozzo P, Fontecave M (2018). Arabidopsis thaliana DGAT3 is a [2fe-2s] protein involved in tag biosynthesis. Sci Rep.

[B4] Bansal S, Durrett TP (2016). Defining the extreme substrate specificity of Euonymus alatus diacylglycerol acetyltransferase, an unusual membrane bound o-acyltransferase. Biosci Rep.

[B5] Brown AP, Kroon JT, Swarbreck D, Febrer M, Larson TR, Graham IA, Caccamo M, Slabas AR (2012). Tissue-specific whole transcriptome sequencing in castor, directed at understanding triacylglycerol lipid biosynthetic pathways. PLoS One.

[B6] Burgal J, Shockey J, Lu C, Dyer J, Larson T, Graham I, Browse J (2008). Metabolic engineering of hydroxy fatty acid production in plants: RcDGAT2 drives dramatic increases in ricinoleate levels in seed oil. Plant Biotechnol J.

[B7] Cagliari A, Margis-Pinheiro M, Loss G, Mastroberti AA, de Araujo Mariath JE, Margis R (2010). Identification and expression analysis of castor bean (Ricinus communis) genes encoding enzymes from the triacylglycerol biosynthesis pathway. Plant Sci.

[B8] Cao H, Shockey JM, Klasson KT, Chapital DC, Mason CB, Scheffler BE (2013). Developmental regulation of diacylglycerol acyltransferase family gene expression in tung tree tissues. PLoS One.

[B9] Chen GQ, Turner C, He X, Nguyen T, McKeon TA, Laudencia-Chingcuanco D (2007). Expression profiles of genes involved in fatty acid and triacylglycerol synthesis in castor bean (Ricinus communis l.). Lipids.

[B10] Chen B, Wang J, Zhang G, Liu J, Manan S, Hu H, Zhao J (2016). Two types of soybean diacylglycerol acyltransferases are differentially involved in triacylglycerol biosynthesis and response to environmental stresses and hormones. Sci Rep.

[B11] Chi X, Hu R, Zhang X, Chen M, Chen N, Pan L, Wang T, Wang M, Yang Z, Wang Q (2014). Cloning and functional analysis of three diacylglycerol acyltransferase genes from peanut (Arachis hypogaea l.). PLoS One.

[B12] Durrett TP, McClosky DD, Tumaney AW, Elzinga DA, Ohlrogge J, Pollard M (2010). A distinct DGAT with sn-3 acetyltransferase activity that synthesizes unusual, reduced-viscosity oils in euonymus and transgenic seeds. Proc Natl Acad Sci U S A.

[B13] Dyer JM, Mullen RT (2008). Engineering plant oils as high-value industrial feedstocks for biorefining: The need for underpinning cell biology research. Physiol Plant.

[B14] Earley KW, Haag JR, Pontes O, Opper K, Juehne T, Song K, Pikaard CS (2006). Gateway-compatible vectors for plant functional genomics and proteomics. Plant J.

[B15] Gao H, Gao Y, Zhang F, Liu B, Ji C, Xue J, Yuan L, Li R (2021). Functional characterization of an novel acyl-coa:Diacylglycerol acyltransferase 3-3 (CsDGAT3-3) gene from Camelina sativa. Plant Sci.

[B16] Hallgren J, Tsirigos KD, Pedersen M, Armenteros JJA, Marcatili P, Nielsen H, Krogh A, Winther O (2022). Deeptmhmm predicts alpha and beta transmembrane proteins using deep neural networks. bioRxiv.

[B17] He X, Turner C, Chen GQ, Lin JT, McKeon TA (2004). Cloning and characterization of a cDNA encoding diacylglycerol acyltransferase from castor bean. Lipids.

[B18] Hernández ML, Whitehead L, He Z, Gazda V, Gilday A, Kozhevnikova E, Vaistij FE, Larson TR, Graham IA (2012). A cytosolic acyltransferase contributes to triacylglycerol synthesis in sucrose-rescued Arabidopsis seed oil catabolism mutants. Plant Physiol.

[B19] Jaworski J, Cahoon EB (2003). Industrial oils from transgenic plants. Curr Opin Plant Biol.

[B20] Kalscheuer R, Steinbüchel A (2003). A novel bifunctional wax ester synthase/acyl-CoA:Diacylglycerol acyltransferase mediates wax ester and triacylglycerol biosynthesis in Acinetobacter calcoaceticus ADP1. J Biol Chem.

[B21] Karimi M, De Meyer B, Hilson P (2005). Modular cloning in plant cells. Trends Plant Sci.

[B22] Kim HU, Lee KR, Go YS, Jung JH, Suh MC, Kim JB (2011). Endoplasmic reticulum-located PDAT1-2 from castor bean enhances hydroxy fatty acid accumulation in transgenic plants. Plant Cell Physiol.

[B23] Kroon JT, Wei W, Simon WJ, Slabas AR (2006). Identification and functional expression of a type 2 acyl-CoA:Diacylglycerol acyltransferase (DGAT2) in developing castor bean seeds which has high homology to the major triglyceride biosynthetic enzyme of fungi and animals. Phytochemistry.

[B24] Lee M, Lenman M, Banaś A, Bafor M, Singh S, Schweizer M, Nilsson R, Liljenberg C, Dahlqvist A, Gummeson PO (1998). Identification of non-heme diiron proteins that catalyze triple bond and epoxy group formation. Science.

[B25] Li F, Wu X, Lam P, Bird D, Zheng H, Samuels L, Jetter R, Kunst L (2008). Identification of the wax ester synthase/acyl-coenzyme A: Diacylglycerol acyltransferase WSD1 required for stem wax ester biosynthesis in Arabidopsis. Plant Physiol.

[B26] Li-Beisson Y, Shorrosh B, Beisson F, Andersson MX, Arondel V, Bates PD, Baud S, Bird D, Debono A, Durrett TP (2010). Acyl-lipid metabolism. Arabidopsis Book.

[B27] Liu J, Rice A, McGlew K, Shaw V, Park H, Clemente T, Pollard M, Ohlrogge J, Durrett TP (2015). Metabolic engineering of oilseed crops to produce high levels of novel acetyl glyceride oils with reduced viscosity, freezing point and calorific value. Plant Biotechnol J.

[B28] Livak KJ, Schmittgen TD (2001). Analysis of relative gene expression data using real-time quantitative pcr and the 2(-delta delta c(t)) method. Methods.

[B29] Lunn D, Wallis JG, Browse J (2019). Tri-hydroxy-triacylglycerol is efficiently produced by position-specific castor acyltransferases. Plant Physiol.

[B30] Lunn D, Le A, Wallis JG, Browse J (2020). Castor LPCAT and PDAT1A act in concert to promote transacylation of hydroxy-fatty acid onto triacylglycerol. Plant Physiol.

[B31] Maraschin FDS, Kulcheski FR, Segatto ALA, Trenz TS, Barrientos-Diaz O, Margis-Pinheiro M, Margis R, Turchetto-Zolet AC (2019). Enzymes of glycerol-3-phosphate pathway in triacylglycerol synthesis in plants: Function, biotechnological application and evolution. Prog Lipid Res.

[B32] McLachlan DH, Lan J, Geilfus CM, Dodd AN, Larson T, Baker A, Hõrak H, Kollist H, He Z, Graham I (2016). The breakdown of stored triacylglycerols is required during light-induced stomatal opening. Curr Biol.

[B33] Mihálik D, Lančaričová A, Mrkvová M, Kaňuková Š, Moravčíková J, Glasa M, Šubr Z, Predajňa L, Hančinský R, Grešíková S (2020). Diacylglycerol acetyltransferase gene isolated from Euonymus europaeus L. altered lipid metabolism in transgenic plant towards the production of acetylated triacylglycerols. Life (Basel).

[B34] Orsavova J, Misurcova L, Ambrozova JV, Vicha R, Mlcek J (2015). Fatty acids composition of vegetable oils and its contribution to dietary energy intake and dependence of cardiovascular mortality on dietary intake of fatty acids. Int J Mol Sci.

[B35] Pan X, Siloto RM, Wickramarathna AD, Mietkiewska E, Weselake RJ (2013). Identification of a pair of phospholipid: Diacylglycerol acyltransferases from developing flax (Linum usitatissimum L.) seed catalyzing the selective production of trilinolenin. J Biol Chem.

[B36] Regmi A, Shockey J, Kotapati HK, Bates PD (2020). Oil-producing metabolons containing DGAT1 use separate substrate pools from those containing DGAT2 or PDAT. Plant Physiol.

[B37] Reynolds KB, Taylor MC, Cullerne DP, Blanchard CL, Wood CC, Singh SP, Petrie JR (2017). A reconfigured Kennedy pathway which promotes efficient accumulation of medium-chain fatty acids in leaf oils. Plant Biotechnol J.

[B38] Saha S, Enugutti B, Rajakumari S, Rajasekharan R (2006). Cytosolic triacylglycerol biosynthetic pathway in oilseeds. Molecular cloning and expression of peanut cytosolic diacylglycerol acyltransferase. Plant Physiol.

[B39] Sandager L, Gustavsson MH, Ståhl U, Dahlqvist A, Wiberg E, Banas A, Lenman M, Ronne H, Stymne S (2002). Storage lipid synthesis is non-essential in yeast. J Biol Chem.

[B40] Shockey JM, Gidda SK, Chapital DC, Kuan JC, Dhanoa PK, Bland JM, Rothstein SJ, Mullen RT, Dyer JM (2006). Tung tree DGAT1 and DGAT2 have nonredundant functions in triacylglycerol biosynthesis and are localized to different subdomains of the endoplasmic reticulum. Plant Cell.

[B41] Shockey J, Lager I, Stymne S, Kotapati HK, Sheffield J, Mason C, Bates PD (2019). Specialized lysophosphatidic acid acyltransferases contribute to unusual fatty acid accumulation in exotic Euphorbiaceae seed oils. Planta.

[B42] Siloto RM, Truksa M, He X, McKeon T, Weselake RJ (2009). Simple methods to detect triacylglycerol biosynthesis in a yeast-based recombinant system. Lipids.

[B43] Sparkes IA, Runions J, Kearns A, Hawes C (2006). Rapid, transient expression of fluorescent fusion proteins in tobacco plants and generation of stably transformed plants. Nat Protoc.

[B44] Sparkes I, Tolley N, Aller I, Svozil J, Osterrieder A, Botchway S, Mueller C, Frigerio L, Hawes C (2010). Five Arabidopsis reticulon isoforms share endoplasmic reticulum location, topology, and membrane-shaping properties. Plant Cell.

[B45] Tran TNT, Breuer RJ, Avanasi Narasimhan R, Parreiras LS, Zhang Y, Sato TK, Durrett TP (2017). Metabolic engineering of Saccharomyces cerevisiae to produce a reduced viscosity oil from lignocellulose. Biotechnol Biofuels.

[B46] Tran TNT, Shelton J, Brown S, Durrett TP (2017). Membrane topology and identification of key residues of EaDAcT, a plant MBOAT with unusual substrate specificity. Plant J.

[B47] Turchetto-Zolet AC, Maraschin FS, de Morais GL, Cagliari A, Andrade CM, Margis-Pinheiro M, Margis R (2011). Evolutionary view of acyl-coa diacylglycerol acyltransferase (DGAT), a key enzyme in neutral lipid biosynthesis. BMC Evol Biol.

[B48] Turchetto-Zolet AC, Christoff AP, Kulcheski FR, Loss-Morais G, Margis R, Margis-Pinheiro M (2016). Diversity and evolution of plant diacylglycerol acyltransferase (DGATS) unveiled by phylogenetic, gene structure and expression analyses. Genet Mol Biol.

[B49] Tvrzicka E, Kremmyda LS, Stankova B, Zak A (2011). Fatty acids as biocompounds: Their role in human metabolism, health and disease--a review. Part 1: Classification, dietary sources and biological functions. Biomed Pap Med Fac Univ Palacky Olomouc Czech Repub.

[B50] van Erp H, Bates PD, Burgal J, Shockey J, Browse J (2011). Castor phospholipid:Diacylglycerol acyltransferase facilitates efficient metabolism of hydroxy fatty acids in transgenic Arabidopsis. Plant Physiol.

[B51] Vernet T, Dignard D, Thomas DY (1987). A family of yeast expression vectors containing the Phage F1 intergenic region. Gene.

[B52] Wu FH, Shen SC, Lee LY, Lee SH, Chan MT, Lin CS (2009). Tape-Arabidopsis sandwich - a simpler Arabidopsis protoplast isolation method. Plant Methods.

[B53] Yen CL, Stone SJ, Koliwad S, Harris C, Farese RV (2008). Glycerolipids. DGAT enzymes and triacylglycerol biosynthesis. J Lipid Res.

[B54] Yoo SD, Cho YH, Sheen J (2007). Arabidopsis mesophyll protoplasts: A versatile cell system for transient gene expression analysis. Nat Protoc.

[B55] Yurchenko O, Shockey JM, Gidda SK, Silver MI, Chapman KD, Mullen RT, Dyer JM (2017). Engineering the production of conjugated fatty acids in arabidopsis thaliana leaves. Plant Biotechnol J.

[B56] Zhang X, Henriques R, Lin SS, Niu QW, Chua NH (2006). Agrobacterium-mediated transformation of Arabidopsis thaliana using the floral dip method. Nat Protoc.

