# Supplementary material for: Functional analysis of alternative castor bean DGAT enzymes
Source: Genet Mol Biol. 2022 Dec 9;46(1 Suppl 1):e20220097. doi: 10.1590/1678-4685-GMB-2022-0097 (PMC9747089; doi:10.1590/1678-4685-GMB-2022-0097)
Supplement: Table S1 - [file 1415-4757-GMB-46-1-s1-e20220097-s1.pdf]

Genetics and Molecular Biology
https://doi.org/10.1590/1678-4685-GMB-2022-0097

Supplementary Material to "Functional analysis of alternative castor bean DGAT enzymes"

Table S1 - Primers used for RT-qPCR.

Name	Sequence	Amplicon size
Rc_DAcTAf<br>Rc_DAcTAr	5'-CGGGTTTGATGCATGAGATT-3'<br>5'-TGAGGGAAAAACAGCCAAAC-3'	214
Rc_DAcTBf<br>Rc_DAcTBr	5'-GTGAAGGGCAGATGGAGGT-3'<br>5'-CCATGAGGTTCCATCTTCTACC-3'	202
Rc_DAcTCf<br>Rc_DAcTCr	5'-CGTGGGAAATCACTTGGTTC-3'<br>5'-GACGGAAAGAAGAGCCAAAA-3'	163
Rc_DAcTDf<br>Rc_DAcTDr	5'-ATTCCTGACCATTGGATTCG-3'<br>5'-TCCAGAAGCCCGTTACAGTT-3'	152
Rc_DGAT3f<br>Rc_DGAT3r	5'-CCTTGTCTGAGGACTTTTCCA-3'<br>5'-TCTCCTTTGCTCGCAATTCT-3'	126
Rc_UBIf<br>Rc_UBIr	5'-ATCGATCGAATCAAGGAACG-3'<br>5'-CACCCTCAATGTTGTAGTCACG-3'	124
Rc_EF1αf<br>Rc_EF1αr	5'-CTGGTGGTTTTGAAGCTGGT-3'<br>5'-AGGTGGGATATAACCCCGAC-3'	200
