# Supplementary material for: Functional analysis of alternative castor bean DGAT enzymes
Source: Genet Mol Biol. 2022 Dec 9;46(1 Suppl 1):e20220097. doi: 10.1590/1678-4685-GMB-2022-0097 (PMC9747089; doi:10.1590/1678-4685-GMB-2022-0097)
Supplement: Table S2 - [file 1415-4757-GMB-46-1-s1-e20220097-s2.pdf]

Genetics and Molecular Biology
https://doi.org/10.1590/1678-4685-GMB-2022-0097

Supplementary Material to "Functional analysis of alternative castor bean DGAT enzymes"

Table S2 - Primers used for cloning castor bean (*Ricinus communis* L.) *DGAT3* and *DAcTA* CDS.

Name	Sequence
Rc_DGAT3_TOPOf	5'-CACCATGGAAGTCTCAGGCCTG-3'
Rc_DGAT3_STOPr	5'-TTAAGATGCAGGGGCCAGACC-3'
Rc_DGAT3_BamHIf	5'-CACCGGATCCATGGAAGTCTCAGGC-3'
Rc_DGAT3_noStop_XbaIr	5'-GGTCTAGAGATGCAGGGGCCAGACCCAA-3'
Rc_DAcTA_BamHIf	5'-CACCGGATCCATGGAAGGAGAGTTCAAGAA-3'
Rc_DAcTA_noStop_XbaIr	5'-GGTCTAGACTTTGTATTCCATTGAAGA-3'
Rc_DAcTA_XbaIr	5'-GGTCTAGATTATTGTATTCCATTGAAGA-3'

Restriction enzyme sites are underlined in the primer sequences.
