# Supplementary material for: Functional analysis of alternative castor bean DGAT enzymes
Source: Genet Mol Biol. 2022 Dec 9;46(1 Suppl 1):e20220097. doi: 10.1590/1678-4685-GMB-2022-0097 (PMC9747089; doi:10.1590/1678-4685-GMB-2022-0097)
Supplement: Figure S1 - [file 1415-4757-GMB-46-1-s1-e20220097-s3.pdf]

Genetics and Molecular Biology
https://doi.org/10.1590/1678-4685-GMB-2022-0097

Supplementary Material to "Functional analysis of alternative castor bean DGAT enzymes"

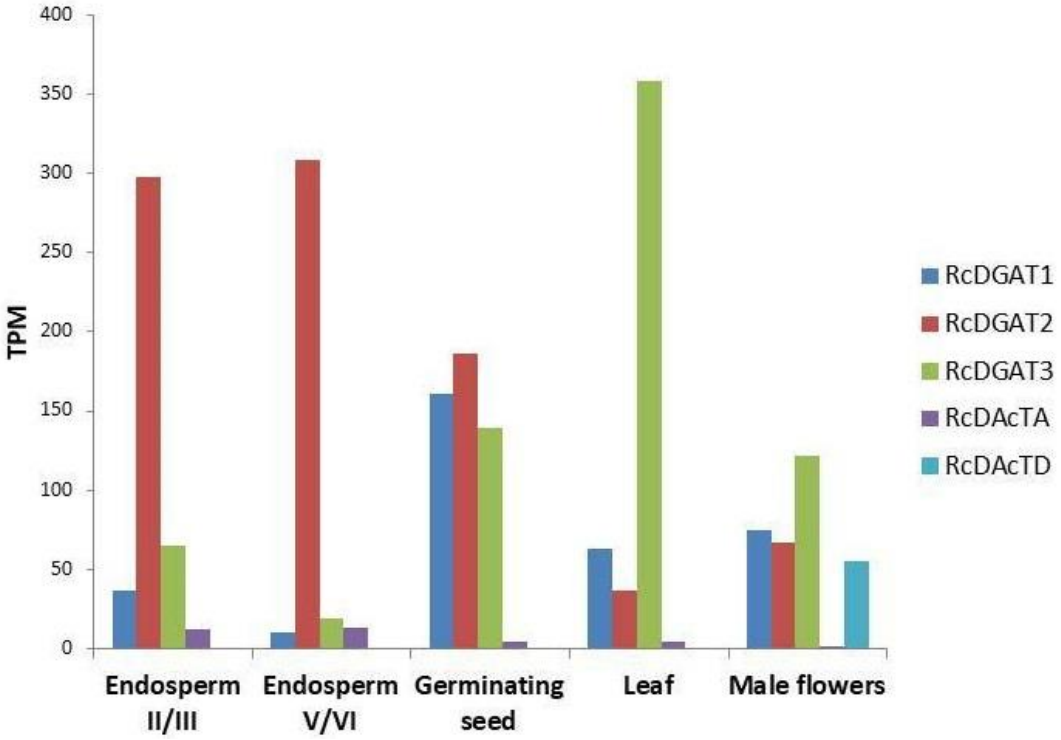

Figure S1 –Transcripts Per Kilobase Million (TPM) of castor bean DGAT genes in different tissues. Data based on the transcriptome of Brown et al. (2012). TPM for *RcDAcTB* and *RcDAcTC* are zero for most tissues, with exception of leaves, which are close to one.
