# Supplementary material for: Functional analysis of alternative castor bean DGAT enzymes
Source: Genet Mol Biol. 2022 Dec 9;46(1 Suppl 1):e20220097. doi: 10.1590/1678-4685-GMB-2022-0097 (PMC9747089; doi:10.1590/1678-4685-GMB-2022-0097)
Supplement: Figure S2 - [file 1415-4757-GMB-46-1-s1-e20220097-s4.pdf]

Genetics and Molecular Biology
https://doi.org/10.1590/1678-4685-GMB-2022-0097

# Supplementary Material to "Functional analysis of alternative castor bean DGAT enzymes"

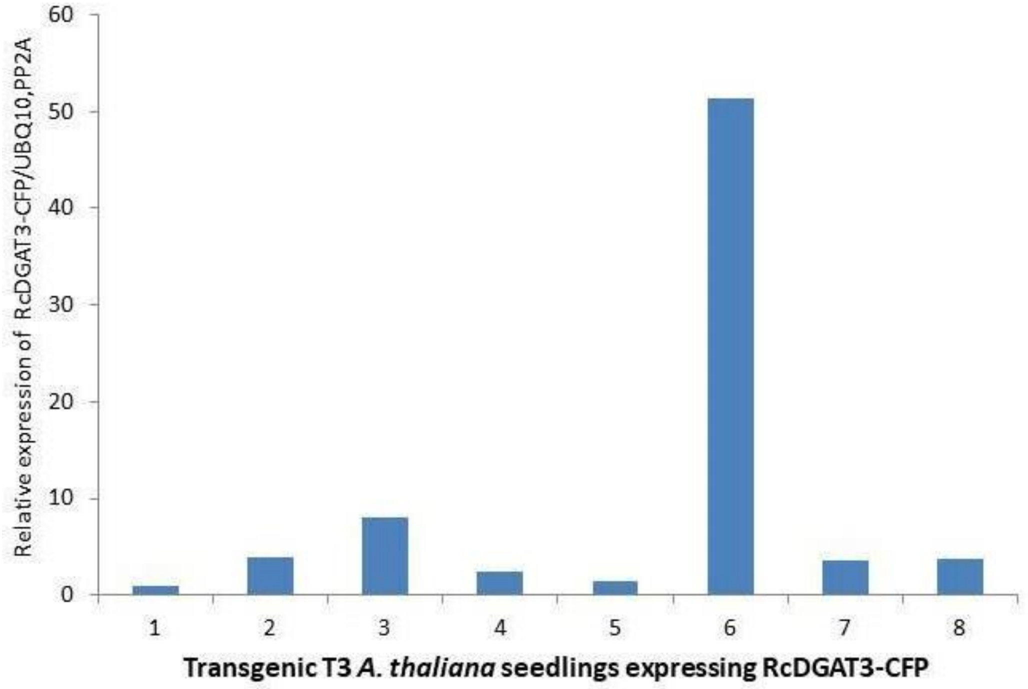


**Figure S2 -** Relative expression of RcDGAT3-CFP in transgenic T3 *A. thaliana* seedlings. Each number represents a different line. *AtUBQ10* and *AtPP2A* were used as reference genes for RT-qPCR.
