# Supplementary material for: Functional analysis of alternative castor bean DGAT enzymes
Source: Genet Mol Biol. 2022 Dec 9;46(1 Suppl 1):e20220097. doi: 10.1590/1678-4685-GMB-2022-0097 (PMC9747089; doi:10.1590/1678-4685-GMB-2022-0097)
Supplement: Figure S3 - [file 1415-4757-GMB-46-1-s1-e20220097-s5.pdf]

Genetics and Molecular Biology
https://doi.org/10.1590/1678-4685-GMB-2022-0097

Supplementary Material to "Functional analysis of alternative castor bean DGAT enzymes"

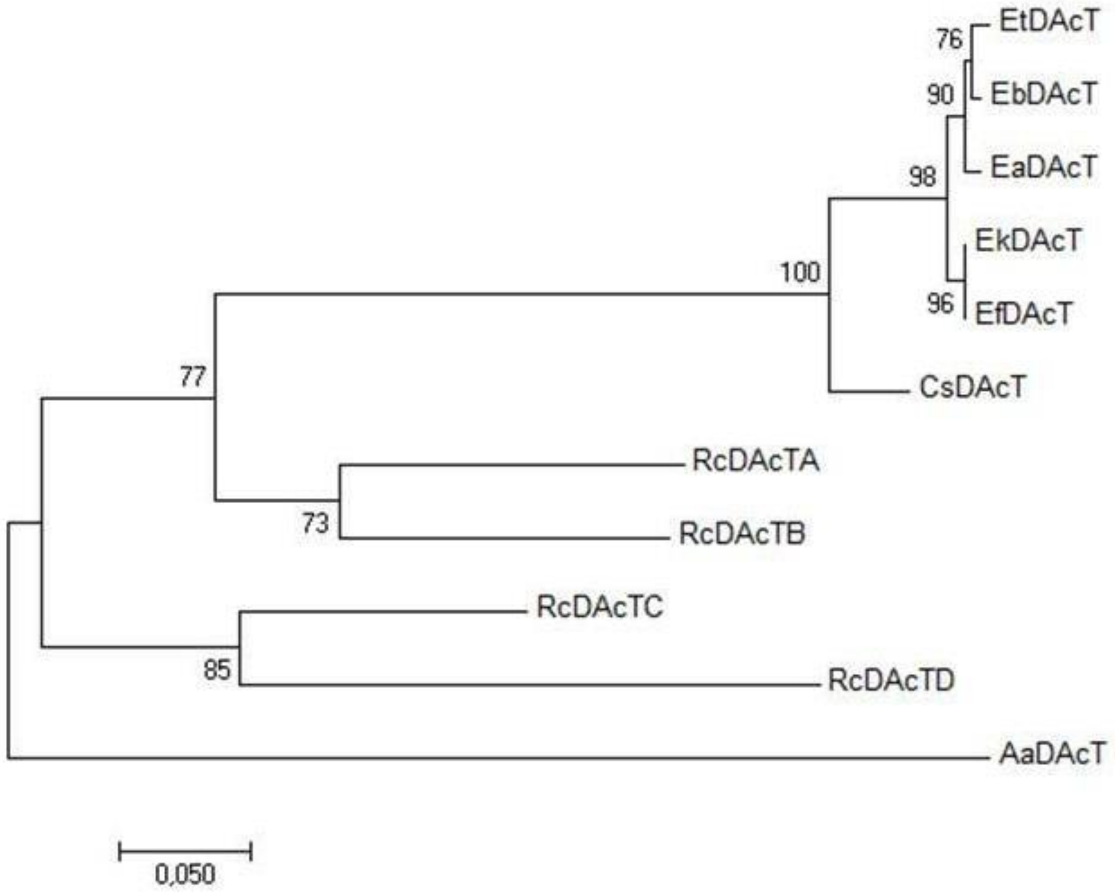

Figure S3 – Maximum Likelihood tree using castor bean putative DAcT CDS sequences and other acetyl-TAG producing plant sequences.ML tree was performed on molecular Evolutionary Analysis 7 (MEGA7) and the original tree is displayed. AaDacT: *Adanis aestivalis*; EtDAcT: *Euonymus atropurpureus*; CsDAcT: *Celastrus scandens*; EbDAcT: *Euonymus maackil*; EkDAcT: *Euonymus klaustschovicus*; EfDAcT: *Euonymus fortune*; EaDAcT: *Euonymus alatus*; RcDAcTA-D: *Ricinus communis*.
